# Long non-coding RNA TSPEAR Antisense RNA 2 is downregulated in rheumatoid arthritis and inhibits the apoptosis of fibroblast-like synoviocytes by downregulating microRNA-212-3p (miR-212-3p)

**DOI:** 10.1080/21655979.2021.2021347

**Published:** 2022-02-03

**Authors:** Zhifen Lv, Shibao Ye, Zhiwen Wang, Panpan Xin, Yuhang Chen, Zhiming Tan, Yu Zhuang

**Affiliations:** Department of Rhumatology and Immunology, Huizhou Municipal Central Hospital, Huizhou, PR. China

**Keywords:** Rheumatoid arthritis, lncRNA TSPEAR-AS2, miR-212-3p, fibroblast-like synoviocytes, apoptosis

## Abstract

Long non-coding RNA (lncRNA) TSPEAR-AS2 (TSPEAR Antisense RNA 2) participates in many human diseases, while its roles in rheumatoid arthritis (RA) are unknown. Plasma expression levels of TSPEAR-AS2 and microRNA (miR)-212-3p in both RA patients and healthy controls were measured by RT-qPCR. Diagnostic potentials of plasma TSPEAR-AS2 and miR-212-3p were assessed by ROC curve analysis. Normalized expression levels of TSPEAR-AS2 and miR-212-3p were subjected to Pearson’s correlation coefficient to evaluate their corrections. TSPEAR-AS2 was significantly downregulated in RA patients, while plasma expression levels of miR-212-3p were significantly increased in RA patients. The expression of TSPEAR-AS2 and miR-212-3p showed promising diagnostic value for RA. Plasma expression levels of TSPEAR-AS2 and miR-212-3p were significantly and inversely correlated in RA patients but not in healthy controls. Besides, overexpression of TSPEAR-AS2 decreased the apoptosis of RA HFLSs, while miR-212-3p increased cell apoptosis. In addition, miR-212-3p attenuated the effects of overexpression of TSPEAR-AS2. Overexpression of TSPEAR-AS2 decreased the expression levels of miR-212-3p in HFLS, while overexpression of miR-212-3p did not affect the expression of TSPEAR-AS2. In conclusion, TSPEAR-AS2 is downregulated in RA and its overexpression can decrease the apoptosis of RA HFLSs by downregulating miR-212-3p.

## Background

Rheumatoid arthritis (RA) is a common autoimmune disorder that affects joints [1]. RA affects more than 1% of the population during their lifetime [2]. Due to the lack of effective treatment strategies and high recurrence rate, RA has become a heavy burden of public healthy [3,4]. With the efforts that have been made on the treatment of RA, sustained remission is now common in RA patients at early stages [5]. However, most patients with RA were diagnosed at advanced stages and pharmacologic therapy is usually required during their whole lifetime [6], bringing heavy burdens on the families of patients.

RA is characterized by the promoted proliferation and inhibited apoptosis of human fibroblast-like synoviocytes (HFLS) [7]. HFLS produces inflammatory cytokines and chemokines to aggregate inflammatory responses in RA [7]. Besides that, matrix-degrading molecules secreted by HFLS cause progressive joint destruction and synovial hyperplasia [7]. Therefore, inducing the apoptosis of HFLS is a key target for the clinical treatment of RA [8,9]. MiR-212-3p can inhibit the proliferation, and induce the apoptosis of HFLS by inhibiting SOX5, which in turn improves RA [10]. Long non-coding RNA (lncRNA) TSPEAR-AS2 (TSPEAR Antisense RNA 2) is a novel lncRNA that promotes oral cancer and gastric cancer [11,12]. Our preliminary transcriptome analysis showed that TSPEAR-AS2 was downregulated in RA and was inversely correlated with miR-212-3p. Therefore, TSPEAR-AS2 may interact with miR-212-3p to participate in RA. We then explored the role of TSPEAR-AS2 in RA and the results showed that it was downregulated in RA and inhibited the apoptosis of HFLS possibly by downregulating miR-212-3p.

## Materials and methods

### Research subject

This study included 73 patients with RA (stage 3) and 66 healthy volunteers who were admitted at Huizhou Municipal Central Hospital from March 2015 to March 2018. All RA patients were diagnosed according to standard criteria. This study excluded other clinical disorders from all patients. All 66 healthy volunteers received systemic physiological examinations and all indicators were within the normal range. The 73 RA patients included 40 males and 33 females, and the age range is 30–66 years old with the mean age of 47.9 ± 5.7 years old. The 66 healthy volunteers included 35 males and 31 females, and age range is 32–68 years old with the mean age of 48.5 ± 5.2 years old. No significant differences in age, gender and other clinical data were found between the two groups. This study was approved by the Ethics Committee of Huizhou Central People’s Hospital. All patients and healthy controls signed the informed consent.

### Specimens and cells

Fasting blood (5 ml) was obtained from each participant to isolate plasma samples through conventional method. Fibroblast-like synoviocytes (HFLSs) were isolated from 2 RA patients and one healthy control using the method reported by Lee *et al*. [13]. HFLSs were cultivated and subsequent experiments were performed using cells collected from passage 3 to 5. HFLSs used in the present study were verified by detecting specially expressed (vimentin) and non-expressed (CD68) genes through immunohistochemistry (IHC).

### RNA extractions and real-time quantitative PCR (RT-qPCR)

Total RNAs were extracted, followed by qPCRs to detect the expression levels of TSPEAR-AS2 using 18S rRNA as the internal control. All-in-One^TM^ miRNA qRT-PCR Detection Kit (Genecopoeia) was used to detect the expression levels of miR-212-3p with U6 as the internal control. Ct values were normalized using the 2^−ΔΔCT^ method.

### Cell transfection

Cells were overexpressed with TSPEAR-AS2 or miR-212-3p by transfecting pcDNA3.1-TSPEAR-AS2 expression vector or hsa-miR-212-3p mimic (Invitrogen) through Lipofectamine 2000 (Invitrogen)-mediated transfections. All steps were completed following the manufacturer’s instructions.

### Cell apoptosis assay

Cells were cultivated in a 6-well plate (50,000 cells in 2 ml non-serum medium) for 24 h. After 0.25% trypsin digestion, Annexin V-FITC (Dojindo, Japan) and propidium iodide (PI) was performed. Finally, flow cytometry was applied for cell apoptosis analysis.

### Statistical analysis

Unpaired t test and ANOVA Tukey test were used to compare two and multiple groups, respectively. Pearson’s correlation coefficient was used to analyze correlations. Diagnostic potentials of plasma expression levels of TSPEAR-AS2 and miR-212-3p for RA were analyzed by ROC curve. Differences were statistically significant at *p* < 0.05.

## Results

### The expression of TSPEAR-AS2 and miR-212-3p were altered in RA patients

**Figure 1.**
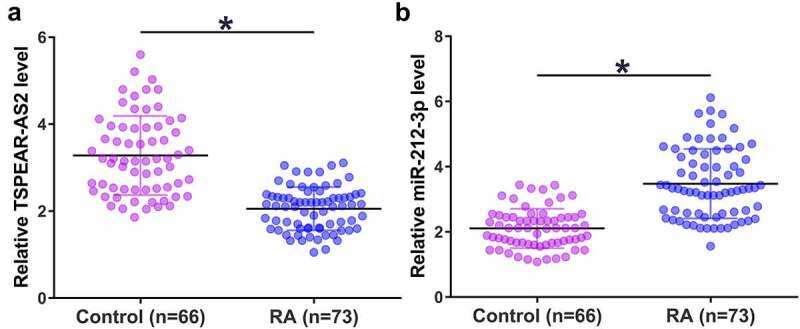
TSPEAR-AS2 and miR-212-3p showed altered expression pattern in RA patients. RT-qPCR results showed that plasma TSPEAR-AS2 was significantly downregulated (a), while plasma miR-212-3p was upregulated (b) in RA patients than that in healthy controls (*, *p* < 0.05).

Altered gene expression could indicate gene function. The expression levels of TSPEAR-AS2 and miR-212-3p in plasma from patient and the healthy controls were detected by performing RT-qPCR. The data showed that TSPEAR-AS2 was obviously downregulated in RA plasma samples (Figure 1a), while miR-212-3p was significantly downregulated in RA plasma samples (Figure 1b) compared to that in the control plasma samples (*p* < 0.05). These results indicated that TSPEAR-AS2 and miR-212-3p may participate in RA.

### TSPEAR-AS2 and miR-212-3p showed diagnostic values for RA

**Figure 2.**
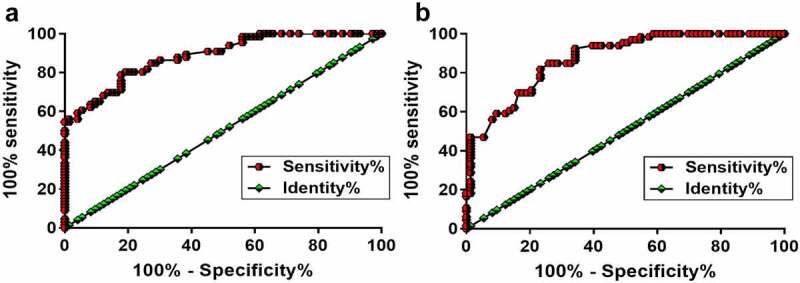
Altered plasma expression levels of TSPEAR-AS2 and miR-212-3p distinguished RA patients from healthy controls. ROC curve analysis showed that altered plasma expression levels of TSPEAR-AS2 (a) and miR-212-3p (b) distinguished RA patients from healthy controls.

ROC curve was applied to study the diagnostic value of TSPEAR-AS2 and miR-212-3p for RA (Figure 2). TSPEAR-AS2 provided an area under the curve (AUC) of 0.89 (standard error: 0.027; 95% CI: 0.83–0.94, Figure 2a). For miR-212-3p, AUC was 0.87 (standard error: 0.028; 95% CI: 0.82–0.93, Figure 2b). These results indicated that TSPEAR-AS2 and miR-212-3p might be utilized to improve the diagnosis of RA.

### TSPEAR-AS2 and miR-212-3p were closely correlated with each other

**Figure 3.**
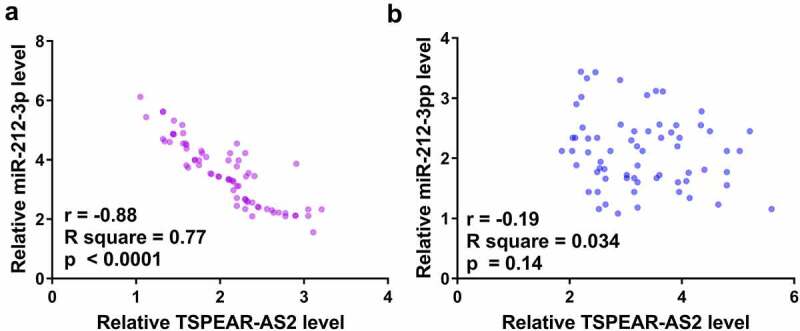
Plasma expression levels of TSPEAR-AS2 and miR-212-3p were significantly and inversely correlated in RA patients but not in healthy controls. Pearson’s correlation coefficient showed that plasma levels of TSPEAR-AS2 and miR-212-3p were significantly correlated in RA patients (a), but not in healthy controls (b).

Correlations can indicate gene interactions. Pearson’s correlation coefficient illustrated that the expression levels of TSPEAR-AS2 and miR-212-3p were closely correlated across RA samples (Figure 3a). However, the correlation between plasma expression levels of TSPEAR-AS2 and miR-212-3p was not significantly correlated in the healthy controls (Figure 3b). Therefore, SPEAR-AS2 and miR-212-3p may interact with each other in RA.

### Overexpression of TSPEAR-AS2 decreased the expression levels of miR-212-3p in HFLS

**Figure 4.**
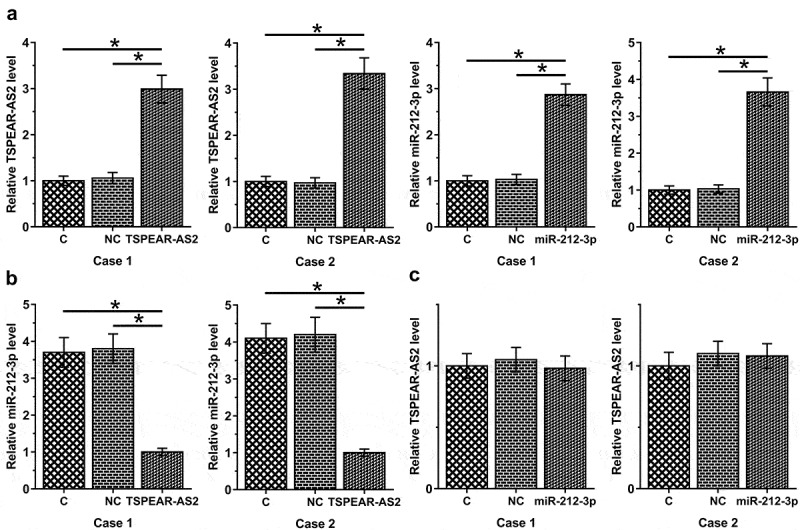
Overexpression of TSPEAR-AS2 led to inhibited expression of miR-212-3p in HFLS. After transfection, overexpression of TSPEAR-AS2 and miR-212-3p were achieved in HFLS isolated from 2 RA patients (a). Compared with control (c) and negative control (NC) groups, overexpression of TSPEAR-AS2 led to downregulation of miR-212-3p (b). However, overexpression of miR-212-3p did not affect the expression of TSPEAR-AS2 (C) (*, *p* < 0.05).

To further explore the potential interactions between TSPEAR-AS2 and miR-212-3p in RA, overexpression of TSPEAR-AS2 or miR-212-3p was achieved in HFLS isolated from 2 RA patients (Figure 4a, *p* < 0.05). No significant difference in the expression levels of TSPEAR-AS2 or miR-212-3p was observed between C and NC groups, suggesting that transfection alone did not affect the expression of TSPEAR-AS2 and miR-212-3p. Compared with the control (C) and negative control (NC) groups, overexpression of TSPEAR-AS2 led to downregulation of miR-212-3p (Figure 4b, *p* < 0.05). However, overexpression of miR-212-3p did not affect the expression of TSPEAR-AS2 (Figure 4c).

### TSPEAR-AS2 overexpression decreased the apoptosis of HFLS through miR-212-3p

**Figure 5.**
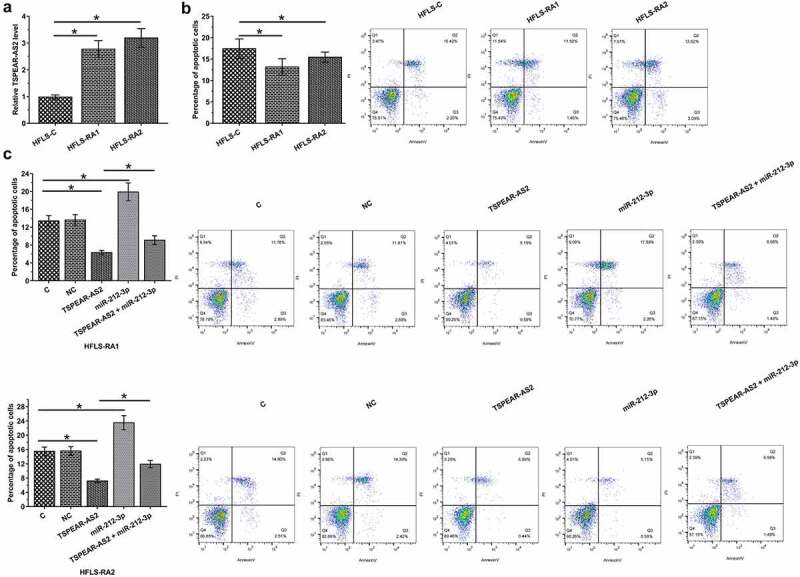
Overexpression of TSPEAR-AS2 inhibited the apoptosis of HFLS through miR-212-3p. The expression of TSPEAR-AS2 in two cases of HFLS from RA patients (HFLS-RA1 and HFLS-RA2) and one case of HFLS from healthy control (HFLS-C) were analyzed by RT-qPCR. It was observed that the expression levels of TSPEAR-AS2 in HFLS-RA1 and HFLS-RA2 were significantly higher compared to that in HFLS-C (a). Cell apoptosis analysis showed that the apoptotic rates of HFLS-RA1 and HFLS-RA2 were significantly lower in comparison to that in HFLS-C (b). Overexpression of TSPEAR-AS2 led to inhibited, while miR-212-3p led to promoted apoptosis of HFLS isolated from 2 RA patients. In addition, miR-212-3p attenuated the enhancing effects of overexpression of TSPEAR-AS2 on cell apoptosis (c) (*, *p* < 0.05).

The expression of TSPEAR-AS2 in two cases of HFLS from RA patients (HFLS-RA1 and HFLS-RA2) and one case of HFLS from healthy control (HFLS-C) was analyzed by RT-qPCR. It showed that the expression levels of TSPEAR-AS2 in HFLS-RA1 and HFLS-RA2 were significantly higher compared to that in HFLS-C (Figure 5a, *p* < 0.05). Cell apoptosis analysis showed that the apoptotic rates of HFLS-RA1 and HFLS-RA2 were significantly lower in comparison to that in HFLS-C (Figure 5b, *p* < 0.05). Overexpression of TSPEAR-AS2 decreased the apoptosis of RA HFLSs, while miR-212-3p increased the apoptosis of RA HFLSs (Figure 5c, *p* < 0.05). Moreover, miR-212-3p suppressed the role of TSPEAR-AS2 in cell apoptosis (*p* < 0.05). In addition, BrdU assay was also performed to explore the role of TSPEAR-AS2 in cell proliferation (10% FBS). No obvious effects of TSPEAR-AS2 on cell proliferation was observed (data not shown). These results indicated that overexpression of TSPEAR-AS2 decreased the apoptosis of HFLS through miR-212-3p.

## Discussion

The pathogenesis and molecular mechanisms of RA are still largely unknown, makes it challenge in the clinical treatment of this disease. The key findings of the present study are that TSPEAR-AS2 downregulated in RA and overexpression of TSPEAR-AS2 may inhibit the apoptosis of HFLS through the downregulation of miR-212-3p, which in turn promotes the development of RA.

RA is a common arthritic disease that can easily be misdiagnosed in clinical practice [14].

Accurate diagnosis and effective treatment are important for the patient’s prognosis. In a recent study, Liu *et al*. reported the involvement of miR-212-3p in RA patients. However, this study did not explore the clinical application values of miR-212-3p in the diagnosis of RA. We reported the upregulation of miR-212-3p in RA patients. ROC curve analysis showed that downregulated plasma expression of circulating miR-212-3p effectively distinguished RA patients from healthy controls, indicating the application potentials of miR-212-3p in the diagnosis of miR-212-3p.

Previous studies have shown that the expression of miR-212-3p in some pathological processes, such as the development and progression of osteosarcoma can be regulated by lncRNAs [15,16]. Interestingly, our study found that TSPEAR-AS2 is likely an upstream inhibitor of miR-212-3p in HFLS, which is a key player in the pathogenesis of RA [7]. In addition, the inhibition of miR-212-3p by TSPEAR-AS2 is likely involved in the regulation of the apoptosis of HFLS. Therefore, our data support the hypothesis that TSPEAR-AS2 may inhibit the apoptosis of HFLS by inhibiting the expression of miR-212-3p, thereby promoting the development of RA.

It is worth noting that overexpression of miR-212-3p only partially attenuated the inhibitory effects of overexpression of TSPEAR-AS2 on the apoptosis of HFLS, indicating that TSPEAR-AS2 may interact with multiple downstream effectors to participate in the regulation of the apoptosis of HFLS. In addition, significant correlation between plasma expression levels of TSPEAR-AS2 and miR-212-3p were found only in RA patients but not in healthy controls, indicating the existing of pathological factors that mediate the interactions between TSPEAR-AS2 and miR-212-3p in RA. However, this study only included samples of HFLS from 2 patients with RA and 1 healthy control. The sample size is small. Our conclusions should be verified by future studies with cells from more patients and healthy controls. Besides, although the molecular mechanisms of RA and the involvement of ncRNAs in this disease have been widely explored [17,18], more studies are still needed to deeply elucidate the molecular factors that regulate RA.

## Conclusion

In conclusion, TSPEAR-AS2 is downregulated in RA, and it may promote RA by inhibiting the apoptosis of HFLS through the downregulation of miR-212-3p.

## Data Availability

The data that support the findings of this study are available on request from the corresponding author. The data are not publicly available due to their containing information that could compromise the privacy of research participants.

## Research highlights

1. TSPEAR-AS2 was significantly downregulated in RA patients, while plasma miR-212-3p was significantly upregulated in RA patients.
2. Overexpression of TSPEAR-AS2 decreased the apoptosis of RA HFLSs, while miR-212-3p increased cell apoptosis.
3. Overexpression of TSPEAR-AS2 decreased the expression levels of miR-212-3p in HFLS.
